# Effects of Hot-Water Extract from Vine Tea (*Ampelopsis grossedentata*) on Acrylamide Formation, Quality and Consumer Acceptability of Bread

**DOI:** 10.3390/foods9030373

**Published:** 2020-03-23

**Authors:** Qian Ma, Shengbao Cai, Yijia Jia, Xiyan Sun, Junjie Yi, Jiang Du

**Affiliations:** 1 Faculty of Agriculture and Food, Yunnan Institute of Food Safety, Kunming University of Science and Technology, Kunming 650500, China; kmustma95@163.com (Q.M.); caikmust2013@163.com (S.C.); yijia529@163.com (Y.J.); junjieyi@kust.edu.cn (J.Y.); 2 Faculty of Art and Communication, Kunming University of Science and Technology, Kunming 650500, China; sunxiyan_02@163.com

**Keywords:** acrylamide, bread texture, consumer acceptability, dihydromyricetin, polyphenol composition, vine tea

## Abstract

Acrylamide is a harmful substance that could be inhibited by natural products. Vine tea is an edible herb belonging to the *Vitaceae* family and has been approved by Chinese authorities as a new food ingredient in 2013. However, the effects of vine tea extract on acrylamide formation and bread quality are rarely investigated. In this study, the polyphenol composition of hot-water extract from vine tea was characterized by ultrahigh-performance liquid chromatography coupled with high-resolution mass spectrometry (UHPLC-ESI-HRMS/MS), and its effects on acrylamide formation, quality, and consumer acceptability of bread were investigated. Vine tea extract and its main polyphenol, dihydromyricetin, significantly inhibited the acrylamide formation in bread, especially the low dose of vine tea extract (1.25 g/kg), which decreased the acrylamide formation by 58.23%. The color and texture of bread were significantly affected by vine tea extract or dihydromyricetin, whereas the moisture content was not changed remarkably. Triangle and paired preference tests indicated that, although the aroma, appearance, and taste of the bread with vine tea extract significantly differ from those of the control bread, vine tea extract did not significantly affect the consumer acceptability. In conclusion, the addition of vine tea extract could be used to develop a new and healthy bread product with low acrylamide content.

## 1. Introduction

Bread is a staple food in western countries, and breadmaking has been known for over 6000 years [1]. After being baked, the bread can increase its color, taste, and flavor, thus attracting many consumers. Although high-temperature processing can provide good bread flavor, it also produces some chemical hazards, such as heterocyclic aromatic compounds, advanced glycation end products, and acrylamide through Maillard reaction at high temperature [2]. Among them, acrylamide is a neurotoxin and potential carcinogen in humans [3] that is formed predominantly via Maillard reaction between the amino acid asparagine and reducing sugars [3], through reaction between aspartic acid and reducing sugars [4], or through the decarboxylation and deamination of asparagine [5]. Since 2002, the Swedish National Food Administration has detected acrylamide in several heat-treated, carbohydrate-rich foods, such as coffee, bread, potato chips, and crisps, and thus added this chemical into the list of food-borne toxicants [6]. Acrylamide in cooked foods has substantially threatened human health due to its high toxicity and possible biological effects [7].

The modification of raw materials, optimization of processing conditions, and addition of exogenous additives can reduce acrylamide formation [8]. Bråthen et al. [9] found that changing the pH could reduce the acrylamide content during frying and baking. Previous study has also found that different additives, such as extracts of cinnamon, thyme, or green tea, significantly reduced the acrylamide amount in fried potatoes [10]. Adding the antioxidant of bamboo leaves (AOB) effectively reduces acrylamide formation and can be used as a natural antioxidant to reduce acrylamide content in potato crisps and French fries [11]. Plant polyphenols possess excellent antioxidant properties and many other bioactive activities. Mildner-Szkudlarz et al. [12] found that adding at least 0.1% polyphenols in the bread significantly reduced its acrylamide content. Kotsiou et al. [13] also reported that the polyphenols may inhibit acrylamide formation because of their strong antioxidant activity; this effect may be related and directly proportional to the number of hydroxyl groups in the polyphenol benzene ring.

*Ampelopsis grossedentata* is a traditional Chinese edible herb belonging to Vitaceae family and widely distributed in southern China. The leaves and stems of *A. grossedentata* are also called vine tea, which has been regarded as a healthy drink for hundreds of years [14] and approved by Chinese authorities as a new food ingredient in 2013. Studies have indicated that vine tea or its main polyphenolic compounds exhibited significant antioxidant [15], antidiabetic [16], antihyperglycemic [16], and antitumor properties [17]. Polyphenols in vine tea are considered the main active substances. Dihydromyricetin is the most abundant polyphenol found in vine tea with an amount exceeding 30% of the total polyphenolic content [18]. However, the effects of vine tea on acrylamide formation and quality of bread are poorly investigated. Therefore, this study aims to determine the effect of hot-water vine tea extract on the acrylamide formation of baked bread and to further investigate the changes in the physicochemical and antioxidant properties of bread. Moreover, whether the difference of bread is perceived and preferred by consumers was also studied by triangle and paired preference tests.

## 2. Materials and Methods

### 2.1. Chemical and Reagents

Acetonitrile (LC-MS grade), *n*-hexane, and formic acid (HPLC grade) were supplied by Merck (Darmstadt, Germany). Bond Elut QuEChERS AOAC Dispersive SPE kit was purchased from Agilent (Agilent, USA). Acrylamide, acrylamide-d3, and 2-diphenyl-1-picrylhydrazyl (DPPH) radical were supplied by Sigma Aldrich (St. Louis, MO, USA). All commercial standards of phenolic compounds (purity ≥ 98%) were obtained from Chengdu Must Bio-Technology Co., Ltd. (Chengdu, China).

### 2.2. Preparation and Analysis of Vine Tea Extract

Vine tea was obtained from a local market in Kunming City, Yunnan province, China and collected from Sangzhi County, Zhangjiajie City, Hunan Province, China, in September 2019. After being air-dried, the vine tea was transported to Kunming at room temperature, ground into powder with a Lingdan LD-T300 grinder (Shanghai, China), and passed through a 60-mesh sieve. The vine tea powder (500 g) was boiled with 10 L of de-ionized (DI) water (1:20 *m*/*v*) for 1.0 h. After being cooled to room temperature, the slurry was filtered. The filtrate was then concentrated by Heidolph Hei-VAP rotary evaporation concentrator and lyophilized with a lyophilizer (Christ Alpha 1-2 LD plus, Germany). The dry extract was placed at −20 °C for further experiment.

Polyphenols in vine tea extract were qualitatively and quantitatively analyzed using an ultrahigh-performance liquid chromatography (UHPLC) system (Thermo Fisher Ultimate 3000) coupled with a Thermo Fisher Scientific Q-Exactive Orbitrap mass spectrometer (Bremen, Germany) in the negative mode. The separation was performed with an Zorbax SB-C18 column (Agilent, 2.1 mm × 100 mm, 1.7 μm) at 30 °C. Ultrapure water containing 0.1% HPLC-grade formic acid (phase A) and LC-MS-grade acetonitrile (phase B) were applied as mobile phases with the following elution gradient: 0–2 min, 5% B; 2–14 min, 5%–35% B; 14–17 min, 35%–5% B; and 17–20 min, 5% B. The injected volume of sample was 2 μL. Flow rate was set as 0.2 mL/min. The mass condition of spray voltage (3.3 kV), heater temperature (350 °C), auxiliary gas flow (8.0 L/min), sweep gas (4.0 L/min), capillary temperature (320 °C), sheath gas flow rate (32.0 L/min), and S-lens RF level (50%) were set. Polyphenols in vine tea were tentatively or positively characterized by comparing the current mass data with published data or those of commercial standards or data in Massbank data base (http://www.massbank.jp/QuickSearch.html). Identified polyphenols were then quantified or semi-quantified with the external calibration curve of the available standard or a commercial standard with the same aglycone. The polyphenol content of vine tea extract was analyzed three times.

### 2.3. Preparation of Bread Samples

Bread was produced as previously described with slight modification [19]. High-grade flour (1.0 kg) was mixed with white granulated sugar (40 g), salt (20 g), shortening (30 g), and active dry yeast (10 g). Then, 620 g water was added to make the dough. The vine tea extract (at doses of 1.25 or 2.5 g/kg flour) or dihydromyricetin (at doses of 9.97 or 19.94 mg/kg flour, the main polyphenol in vine tea extract) were added to the bread formulation. Five different bread groups were prepared in the present work, namely the control group (without supplement), the low-dose vine tea extract group (1.25 g/kg flour), the high-dose vine tea extract group (2.5 g/kg flour), the low-dose dihydromyricetin group (9.97 mg/kg flour), and the high-dose dihydromyricetin group (19.94 mg/kg flour). All materials in each group were mixed well by using a household dough mixer, kneaded for 15 min for twice, and hand-kneaded for another 5 min. Thereafter, the dough of each group was divided into several equal weight parts (100 g), and all parts were fermented at 40 °C and 80% relative humidity for 1.0 h (SMF-32B, WXSMAG, China). Afterward, the bread was baked in the oven (SMD-603E+B, WXSMAG, China) for 20 min at 200 °C. After being cooled to room temperature, some fresh bread in each group was divided into crust and crumb. Crumb sample was obtained from the center of the bread, and crust sample was obtained from the surface (1.5–2 mm thickness). The fresh crust and crumb were used for moisture, color, texture, and antioxidant analysis. Moreover, part of each crust sample was freeze-dried with a Christ Alpha 1-2 LD plus lyophilizer, ground into powder (passed through 100 mesh sieve), and placed in a desiccator for acrylamide extraction and analysis. The remaining fresh bread in each group was used for sensory analysis. Three batches of bread were prepared for each treatment.

### 2.4. Acrylamide Extraction From Bread Crust

Acrylamide extraction was conducted as previously described [19] with minor modification. Exactly 1 g powder from the abovementioned freeze-dried bread crust was placed in a 50-mL centrifuge tube. Then, 5 mL of n-hexane was added to the tube and subjected to vortex for 20 s. Afterwards, 10 mL of Milli-Q water, 10 mL of LC-MS grade acetonitrile, 4.0 g anhydrous MgSO4, and 0.5 g NaCl were successively added. Acrylamide-d3, as the internal standard, was also added into the tube with the final concentration of 100 μg/L. The mixture was mixed well by using a vortex mixer for 1 min, and then centrifuged at 5,000·*g* for 10 min. After centrifugation, n-hexane layer was removed and the acetonitrile layer was concentrated using vacuum centrifugal concentrator (miVac, SP SCIENTIFIC, America) to 1 mL and transferred to a tube (Agilent Bond Elut QuEChERS AOAC Dispersive SPE kit containing 50 mg primary secondary amine and 150 mg anhydrous MgSO4). The tube was vibrated for 1.0 min and then centrifuged at 5,000·*g* for 2.0 min. The supernatant was gathered and evaporated using a vacuum centrifugal concentrator. Thereafter, 300 μL of LC-MS grade acetonitrile was added, transferred into a vial with glass insert, placed at −20 °C for LC-MS analysis.

### 2.5. LC-MS Analysis for Acrylamide

Extracted acrylamide was analyzed using a Nexera XR UHPLC system equipped with an LCMS-8040 triple quadrupole mass spectrometer (Shimadzu, Japan) in the positive mode. A Zorbax Eclipse PAH column (Agilent, 2.1 × 100 mm, 1.8 μm) was applied to separate the chemicals in each sample at 35 °C. Ultrapure water acidified with 0.1% (*v*/*v*) HPLC-grade formic acid and LC-MS-grade acetonitrile were applied as mobile phases A and B, respectively. The gradients of the mobile phases were as follows: 0–1 min, 3% B; 1–2 min, 3%–7% B; 2–3 min, 7%–3% B; and 3–6 min, 3% B. Flow rate was set to 0.3 mL/min. The injection volume of each sample was 3.0 μL. Multi-response monitoring mode was used for acrylamide analysis. For detection, m/z 72 and 55.2 for acrylamide and m/z 75 and 58.2 for acrylamide-d3 were used with the collision energy of 25 V. The capillary voltage was maintained at 3.5 kV. The gas temperature (350 °C) and gas flow rate (650 L/h) were set. The calibration curve (R^2^ = 0.9995) was made by using acrylamide standard at the same detected condition. Acrylamide was subjected to LC-MS analysis three times.

### 2.6. Color Evaluation

The color properties of L* (lightness), a* (redness), and b* (yellowness) of the crust or crumb of the baked bread in each group were analyzed using a Minolta CHROMA METER CR-400 colorimeter (Konica, Japan). The result is expressed as L* (0 = black, 100 = white), a* (−a* = greenness and + a* = redness), and b* (−b* = blueness, +b* = yellowness). Each measurement was performed in triplicate.

### 2.7. Moisture Analysis

Moisture content of the baked bread crumb was determined as previously described [20] with slight modification. Approximately 8 g of each fresh bread crumb was oven dried at 105 °C to constant weight. The exactly initial and final weights of each bread sample were recorded using an electronic precision balance (BSA124S-CW, Sartorius, Germany). The moisture content of each sample was calculated with the following formula:

moisture content (%) = ((W_i_ − W_f_)/W_i_) × 100 (1)

where *W_i_* is the initial weight of the bread sample and *W_f_* is the final weight of the bread sample after being oven-dried. The result was reported as an average of three measurements.

### 2.8. Texture Analysis

Bread crumb structure was measured by texture profile analysis (TPA) with a TPA analyzer (TA-XTplus12587, UK). The bread crumb was sliced into 20-mm thick slices. A 35-mm wide probe P/1S was used to squeeze the bread with 40% penetration depth. The detailed test parameters of pretest speed, test speed, posttest speed; distance of compression; time; trigger type; and data acquisition rate were set as 2.0, 2.0, and 2.0 mm/s; 10.0 mm (the height of the gel is 25 mm); 10.0 s; auto (force); 10.0 g; and 250 pps, respectively. Several qualitative and structural indicators were measured, including hardness, springiness, cohesiveness, gumminess, chewiness, and resilience. Hardness (g) was represented as the peak force of the first compression cycle. Springiness was obtained by dividing the recorded time from the start of the second area to the second probe reversal by that from the start of the first area and the first probe reversal [19]. Cohesiveness was obtained by dividing the area under the second curve by that under the first curve. Gumminess was calculated by multiplying hardness and cohesiveness. Chewiness was defined as the energy value of the solid sample after chewing and swallowing and was calculated by multiplying gumminess and springiness [21]. Resilience refers to the ratio of the compression energy to the recovery energy during the first compression. All measurements were conducted three times.

### 2.9. DPPH Radical Scavenging Activity of Bread Sample

DPPH radical scavenging activity of each bread sample was evaluated as previously described [22] with slight modification. In brief, 1.0 g of dried powder of crust or crumb was added into 40.0 mL of 80% methanol solution to make the final concentration of each sample at 25.0 mg/ml. The mixture was then ultrasonically extracted for 0.5 h at room temperature and centrifuged at 1000·*g* for 10 min. The supernatant of each sample was gathered and used for the DPPH radical scavenging assay. Exactly 0.5 mL of the supernatant from each sample was mixed well with 2.0 mL of 0.1 mM DPPH radical reagent and then maintained in darkness for 30 min at room temperature. The absorbance of each sample was determined using a SpectraMax M5 microplate reader (Molecular Device, USA) at 517 nm as *A_S_*. The mixture of DPPH radical reagent and 80% methanol was applied as a control (*A_c_*). DPPH radical scavenging ability was calculated using the following formula:

DPPH radical scavenging ratio (%) = ((A_c_ - A_S_)/A_c_) × 100 (2)

Each test was performed three times.

### 2.10. Triangle and Paired Preference Tests

Sensory tests were conducted by using the triangle and paired preference tests as described earlier [23] with slight modification. Two sensory tests were conducted in independent sessions to avoid panelist’s sensory fatigue. Thirty untrained panelists (15 of male; 15 of female) aged 20–55 years were recruited among students and staff of Kunming University of Science and Technology (Kunming, China). All bread samples were placed in nontransparent box marked with three random digit codes and maintained hermetically closed until sensory evaluation.

Three stages were performed in triangle tests. In the first stage, smell was evaluated. Three boxes with different samples, a different one and two identical ones, including control bread, sample bread (added 1.25 g/kg vine tea extract) were provided to the panelists. The samples were provided at a random possible combination (YXX, XXY, XYX, YXY, YYX, or XYY). Thirty panelists were asked to recognize the distinct sample compared with the two other samples in terms of smell. Similarly, vision and taste were evaluated respectively in the second and third stage. The test results were interpreted as previously described [23,24]. More than 15 or 17 correct answers indicated a significant difference between the control and sample breads (added vine tea extract) at a level of 0.05 and 0.01, respectively. In paired preference test, panelists were provided with two different samples, namely the control and sample bread (added 1.25 g/kg vine tea extract), and the panelists were requested to enter their preference and write the reason.

### 2.11. Statistical Analysis

Each measurement was performed three times. The result was expressed as mean ± standard deviation (S.D.). All data were analyzed via one-way ANOVA by using Origin 8.5 software (OriginLab, Northampton, MA, USA), and Tukey’s test was employed to determine significant differences (*p* < 0.05).

## 3. Results and Discussion

### 3.1. Characterization of Main Polyphenols in Vine Tea Extract 

**Figure 1:**
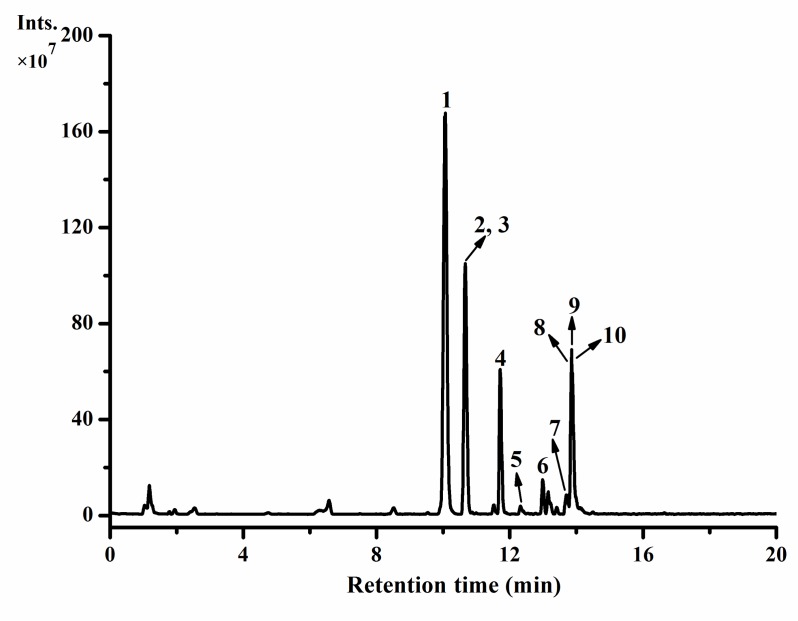
Negative ion current chromatogram of hot-water extract from vine tea (*Ampelopsis grossedentata*): Peaks identification and their MS data are shown in Table 1. Peak 1, dihydromyricetin; peak 2, iso-dihydromyricetin; peak 3, myricetin-3-*O*-glucoside; peak 4, myricetin-3-*O*-rhamnoside; peak 5, quercetin-3-*O*-xyloside; peak 6, quercetin-3-*O*-rhamnoside; peak 7, quercetin; peak 8, phloridzin; peak 9, phloretin; and peak 10, myricetin.

The negative ion current chromatogram is illustrated in Figure 1, and the detailed mass data of the tentatively or positively characterized polyphenols are summarized in Table 1. As shown in Figure 1 and Table 1, 10 main polyphenolic compounds were detected and identified in vine tea extract. Among them, compounds 2 and 5 were tentatively identified as iso-dihydromyricetin (peak 2, [M-H]^-^ m/z = 319.0466) and quercetin-3-*O*-xyloside (peak 5, [M-H]^-^ m/z = 433.0789), respectively, by comparing the current mass data ([M-H]^-^ m/z and MS/MS ion) with those of the corresponding compound reported earlier or Massbank (http://www.massbank.jp/QuickSearch.html). Both compounds have been previously detected in vine tea [14]. For the tentative identification of iso-dihydromyricetin, the mass data of iso-dihydromyricetin from previous studies [14,25,26] and the current work and those of dihydromyricetin in the current work were comprehensive analyzed. The eight other polyphenolic compounds were all positively identified according to the mass data of their corresponding commercial standards. Compound 1 (peak 1, [M-H]^-^ m/z = 319.0467), which was positively identified as dihydromyricetin, showed a relatively higher peak area, indicating that compound 1 was the main polyphenolic compound in the vine tea extract. The quantitative and semiquantitative results of those 10 polyphenolic compounds further proved that compound 1 (dihydromyricetin) was the primary polyphenolic compound in vine tea extract with approximately 7975.01 μg/g of dry weight of extract. This result supports that dihydromyricetin is the main polyphenolic compound in vine tea [27]. Iso-dihydromyricetin was measured as the second largest component of the extract as indicated in Table 1. Myricetin, quercetin, and phloretin detected in the present work have also been previously reported in vine tea [14].

### 3.2. Quantification of Acrylamide with LC-MS

**Figure 2:**
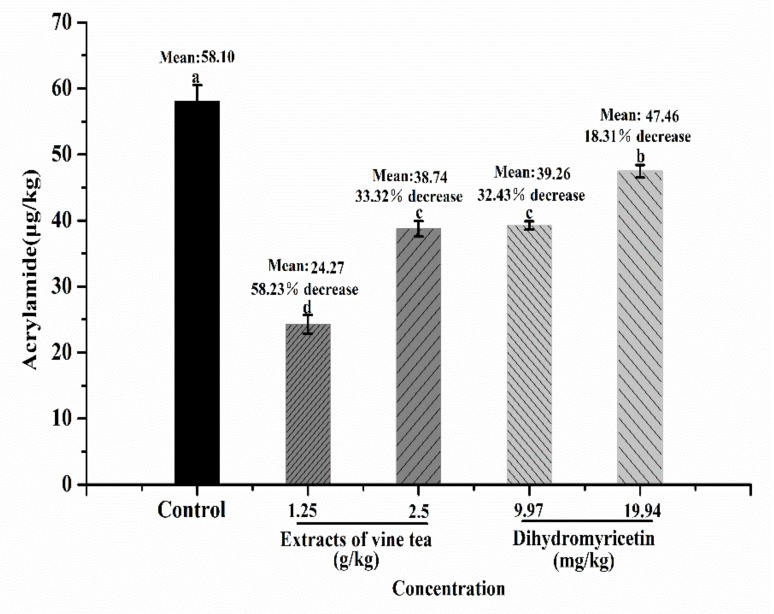
Contents of acrylamide in the crust of sample bread (vine tea extract or dihydromyricetin) and control bread (without supplement): The results are expressed as mean ± SD (*n* = 3). Different letters indicate significant differences (*p* < 0.05).

The formation of acrylamide mainly occurred through the Maillard reaction between asparagine and reducing sugar or carbonyl group [3]. Acrylamide in each bread crust was detected and measured using LC-MS, and the results are summarized in Figure 2. The acrylamide contents of the bread samples added with vine tea extract or its main polyphenol dihydromyricetin were all significantly declined (*p* < 0.05). In the control group, the bread crust had the highest acrylamide content of 58.10 μg/kg, which was within the range of 5−1987 µg/kg for 192 different bread samples reported earlier [28] and similar to the content (52.19 μg/kg) reported by Fu et al. [19]. The acrylamide contents of the 1.25 and 2.5 g/kg vine tea extract groups decreased by 58.23% and 33.32%, respectively, compared with those of the control group. For the 9.97 and 19.94 mg/kg dihydromyricetin groups, 32.43% and 18.31% decreases were observed, respectively. For the low-dose groups, vine tea extract and dihydromyricetin showed better inhibitory effects on the acrylamide formation of bread than their corresponding high-dose groups. This finding was consistent with the study of Mildner-Szkudlarz et al. [12], who reported that quercetin has better inhibitory effect on acrylamide formation in bread at a low dose than at a high dose, and a nonlinear relationship existed between the inhibitory effect and quercetin concentration. This phenomenon may be due to the structure and redox properties of polyphenols. Most polyphenols normally act as an antioxidant to scavenge free radical and/or react with the precursor, intermediates, or acrylamide itself to inhibit acrylamide formation at low concentration [8]. However, high concentration of polyphenolic compounds could produce hydrogen peroxide that participates in the acrylamide formation during bread baking [29]. Moreover, carbonyl moiety, which is also contained in some polyphenolic compounds (i.e. quercetin and rosmarinic acid), may react directly with asparagine in wheat flour to form acrylamide [30], and thereby, addition of high dose of those polyphenols may increase acrylamide formation to some extent. Considering the complexity of the acrylamide formation, the exact mechanisms of how the polyphenols interfere with acrylamide formation need to be further investigated.

In this study, the addition content of dihydromyricetin standard (9.97 or 19.94 mg/kg flour) was equal to the amount of dihydromyricetin contained in vine tea extract in the corresponding concentration group (1.25 or 2.5 g/kg flour, in which dihydromyricetin was 9.97 or 19.94 mg/kg flour) according to the quantitative results of polyphenols (Table 1). However, the inhibitory effect of vine tea extract on acrylamide formation was significantly stronger than that of the corresponding concentration of dihydromyricetin, as shown in Figure 2 (*p* < 0.05). This result indicates that, besides dihydromyricetin, the other polyphenols contained in vine tea extract also contributed to the inhibition of acrylamide formation during bread baking. Komprda et al. [31] reported that the synergism of components (NaHCO_3_/Ca^2+^/citric acid) leads to a possible decrease of acrylamide in gingerbread. However, whether these polyphenols in vine tea extract interact with each other in inhibiting the formation of acrylamide in the present study, such as synergism or antagonism, needs to be further confirmed. In this study, the acrylamide in the 1.25 and 2.5 g/kg vine tea extract groups decreased by 58.23% and 33.32%, respectively. The (−)-epigallocatechin gallate (EGCG) extracted from green tea could inhibit 30.2% to 37.4% of acrylamide with 3.3 g/kg to 9.9 g/kg addition [19]. The difference in inhibitory effect on acrylamide formation may be due to the different polyphenol composition of extracts. Cheng and coauthors [32] have comparatively investigated the different fruit extracts for their activities against acrylamide formation and found that the apple extract has good inhibitory effects. According to the present result and previous findings, it clearly indicates that the polyphenol composition of extract may significantly affect the inhibitory effect on the acrylamide formation.

### 3.3. Color Measurement

Color is an important property that partly determines whether the bread could be accepted by consumers. The color parameters (L*, a*, and b*) of the crust and crumb of all bread samples are summarized in Table 2. For the crust, the lightness (L*) of the crust of sample bread added with vine tea extracts (both doses) or low dose of dihydromyricetin was significantly higher than that of the crust of control bread (*p* < 0.05). All fortification samples, except low-dose vine tea extract (1.25 g/kg), remarkably reduced the a* value of the bread crust compared with the control group (*p* < 0.05). The yellowness (b*) values of the crust samples in both doses of vine tea extract groups significantly decreased compared with that in the control group (*p* < 0.05), while the addition of dihydromyricetin, regardless of concentration, did not dramatically affect the b* values (*p* > 0.05). For the bread crumb, the addition of vine tea extract significantly decreased the values of the L*, a*, and b* compared with the corresponding value of the control bread (*p* < 0.05). Therefore, bread crumb samples containing vine tea extract were darker compared with the control sample. Dihydromyricetin fortification did not significantly affect the values of L* and b* (*p* > 0.05) but significantly reduced the a* values of the bread crumb (*p* < 0.05). EGCG, which is extracted from green tea, significantly increases the L* value of the bread crust but dramatically decreases the L* value of the bread crumb [19]. However, Jing and coauthors [33] found that phenolic-rich extracts from Tartary and common buckwheat significantly increases the L* value of both bread crust and crumb. Moreover, according to previous findings [19,33] and the current data, the changes in a* and b* values are closely associated with materials added in bread, especially the color of added materials, which may profoundly affect the a* and b* values of baked bread. However, the addition of a certain amount of polyphenol extract could significantly increase the L* value of bread crust. In the present work, all sample-added bread crust, except the high-dose dihydromyricetin fortification bread crust, possessed higher L* values in comparison with the control bread crust which is generally believed to be that the added polyphenols inhibit the Maillard reaction of bread and, thereby, gives the bread crust a lighter color. Maillard reaction is related to the color of the bread. It provides the brown color of the bread crust. Although the underlying mechanism of how those polyphenol extracts influence the Maillard reaction of bread is still not very clear, the current data and some previous findings have revealed that polyphenol-rich extracts from different materials were effective to inhibit the Maillard reaction to reduce the acrylamide formation.

### 3.4. Moisture Analysis

The moisture content, which reflects the amount of unbound water in the bread, is a vital quality parameter affecting the shelf life of food. It is found that both the dough nature and the baking process could influence the moisture in bread [34]. As shown in Table 3, the addition of vine tea extract or dihydromyricetin did not significantly affect the moisture contents of the bread when compared with that of the control group (*p* > 0.05). A similar result has been reported in white pan bread with rosemary extract that the addition of rosemary extract did not affect the moisture content of the bread crumb [35]. However, green tea extract that is rich in polyphenols could significantly decrease the moisture content of bread [19,36]. These different results may be caused by different polyphenol composition of the extract. EGCG (main polyphenol in green tea extract) has a large self-association constant and can easily form self-assembled dimers in water, thus mainly accounting for the moisture content decrease of bread [19,37]. Therefore, polyphenols with low self-association constants may have less effect on the moisture content of bread, which needs further studies to confirm.

Moreover, moisture content is closely associated with Maillard reaction and therefore the acrylamide formation of bread. Low moisture content can enhance the acrylamide formation of food material during thermal processing, and a higher relative humidity throughout baking is recommended to, at least partially, avoid acrylamide formation [38]. However, Fu et al. [19] found that both the moisture content and acrylamide amount of the bread significantly decrease when the EGCG, which was extracted from green tea, was added to the bread, and the authors did not explain this phenomenon. In the present study, we found that, although the addition of vine tea extract or dihydromyricetin did not significantly affect the moisture content of bread (*p* > 0.05), it significantly reduced the amount of acrylamide in bread (*p* < 0.05). We hypothesize that the effect of slight moisture change of bread on acrylamide formation may be counteracted and/or replaced by the effect of polyphenol itself on acrylamide formation when polyphenol is added into bread. This hypothesis could be used to explain the paradoxical results observed by Fu et al. [19].

### 3.5. Texture Profile of Bread Crumb

The texture of bread crumb is the most important factor in determining the taste and consumer acceptance of bread, which usually relies on start gelatinization. Six parameters about the bread crumb texture were measured in the present work. As shown in Table 3, compared with the control group, vine tea extract or dihydromyricetin significantly reduced the hardness, gumminess, and chewiness (*p* < 0.05). The springiness of bread with low-dose vine tea extract or dihydromyricetin increased significantly (*p* < 0.05). However, the addition of those samples did not remarkably affect the cohesiveness and resilience (*p* > 0.05). Among those parameters, the hardness is the most considered parameter in bread production, which is related to the bite force and commonly used as an index to determine bread quality [39]. In the present work, the hardness of bread was significantly decreased with increasing ratio of vine tea extract or dihydromyricetin. Similarly, Jing and coauthors [33] reported that bread added with Tartary buckwheat sprout was significantly softer than bread treated with Tartary buckwheat seed, and this phenomenon may be due to the significantly high content of total phenolic compounds in Tartary buckwheat sprout. By contrast, Pasrija et al. [36] reported that microencapsulated green tea polyphenols did not significantly affect the hardness of bread, while Wang et al. [40] found that green tea extract rich in EGCG remarkably increased the hardness of white bread by 40%. The paradoxical results of those different studies about the effect of polyphenols on the hardness of bread may be due to the following: (1) the difference in quality of flour used for bread making, including protein and reducing sugar contents; (2) different bread making and/or baking processes; and (3) the difference in the composition and content of polyphenols added into bread. Changes in gluten network or gluten content could result in the variance of the bread texture [41]. The polyphenols added into bread may affect the gluten interaction in the dough, resulting in changes in the spatial structure of the dough and the texture of the bread [42], and this observation needs further studies to elucidate.

### 3.6. DPPH Radical Scavenging Activity in Bread

**Figure 3:**
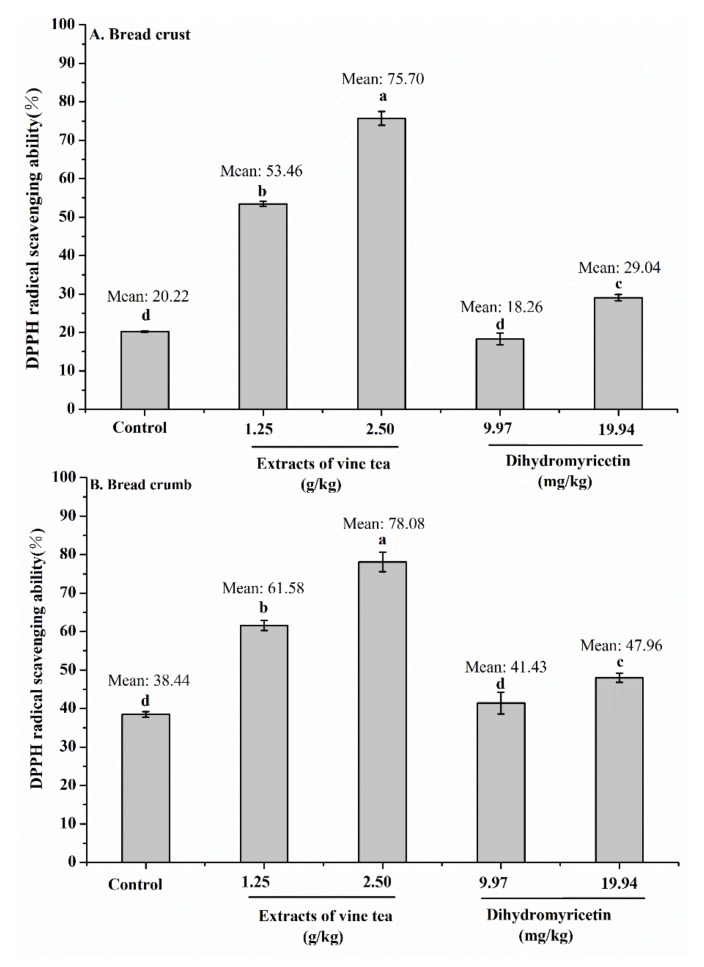
DPPH (2-diphenyl-1-picrylhydrazyl) radical scavenging abilities of the crust (**A**) and crumb (**B**) of the control bread (without supplement) and sample bread (vine tea extract or dihydromyricetin): The results are expressed as mean ± SD (*n* = 3). Different letters indicate significant differences (*p* < 0.05).

Antioxidant activity is a major biological activity of polyphenolic compounds. Scavenging the free radical during bread baking is one mechanism for polyphenolic compounds in inhibiting acrylamide formation in bread [8,19]. Moreover, antioxidants, such as polyphenolic compounds, may prevent lipid oxidation to limit the accumulation of carbonyl groups and thereby inhibit the formation of acrylamide [43]. Considering that the addition of vine tea extract or dihydromyricetin significantly inhibited the acrylamide formation in bread when compared to that in the control bread (*p* < 0.05, Figure 2), the antioxidant activities of the crust and crumb of the bread with or without supplements were comparatively measured by DPPH radical scavenging method. As shown in Figure 3, the DPPH radical scavenging capacities of the crust and crumb of all sample bread, except that with low addition of dihydromyricetin bread, significantly increased when compared with the counterpart of the control bread. This finding may further confirm that the antioxidant activities of vine tea extract or dihydromyricetin were at least partly responsible for inhibiting the acrylamide formation in bread. Similarly, green tea extract could significantly increase the antioxidant activity of sponge cakes [44]. Pasrija and coauthors [36] also found that the bread with added encapsulated powder of green tea extract has a high antioxidant capacity. However, the present study revealed that the addition of low-dose dihydromyricetin did not significantly increase the DPPH radical scavenging capacities of the bread, whether in the crust or crumb. This finding may be due to the low-dose addition and the loss of dihydromyricetin during bread baking via consumption that inhibited acrylamide formation and its thermal decomposition. Fu et al. [19] also found that EGCG is lost at a high rate during bread baking. Moreover, according to the results of DPPH radical scavenging activity and inhibition of acrylamide formation, it clearly indicates that some other mechanism may be involved in polyphenol inhibiting acrylamide formation besides acting as an antioxidant, which needs to be revealed by further experiment.

### 3.7. Overall Quality and Likeability

Although the bread added with low-dose vine tea extract contained the lowest amount of acrylamide (Figure 2), its quality such as color and hardness changed significantly (Table 2; Table 3). Sensory analysis is one of the most direct and effective methods to evaluate the consumer acceptability and overall quality characteristics of a product. Therefore, triangle and paired preference tests were performed to determine whether the addition of vine tea extract in the bread can be perceived and preferred by consumers. As shown in Table 4, triangle test results indicate that the aroma, appearance, and taste of the bread added vine tea extract were significantly different from those of the control bread. The aroma, color, and taste of vine tea extract may be mainly responsible for these differences. In the paired preference test, the preference of panelists between the sample and control bread is not significant difference (Table 4). In the questionnaire survey, some panelists mentioned that the bread added with vine tea extract has a special tea aroma that they preferred, and others chose the control bread because of the color. In general, the addition of vine tea extract did not significantly affect the consumer acceptability of bread. However, if consumers know that the bread with vine tea extract has low acrylamide and high antioxidant property, then they might be more willing to choose the bread with vine tea extract over the control bread.

## 4. Conclusions

The polyphenol composition of hot-water extract from vine tea (*A. grossedentata*) was characterized by UHPLC-ESI-HRMS/MS, and its effects on acrylamide formation, quality, and consumer acceptability of bread were investigated. Ten polyphenolic compounds were tentatively or positively identified in vine tea extract, and dihydromyricetin had the highest content at approximately 7975.01 μg/g of dry weight of extract. Vine tea extract and dihydromyricetin could significantly reduce the acrylamide content in bread, especially the low-dose vine tea extract (1.25 g/kg) that decreased the acrylamide formation by 58.23%. The color and texture, especially the hardness, of bread were significantly affected by the addition of vine tea extract or dihydromyricetin, whereas the moisture content of bread was not changed remarkably. The addition of vine tea extract or high dose of dihydromyricetin significantly increased the DPPH radical scavenging activity of bread. Triangle and paired preference tests revealed that, although the aroma, appearance, and taste of the bread added with vine tea extract were significantly different from those of the control bread, the addition of vine tea extract did not significantly affect the consumer acceptability of bread. These results indicate that the bread added vine tea extract could be developed as a new and healthy bread product.

## Figures and Tables

**Table 1:** Identification and quantification of main polyphenolic compounds in vine tea (*Ampelopsis grossedentata*) extract.

Peak No.	Compounds	Formula	RT^*^(min)	[M-H]^−^ (m/z)	Fragments (MS/MS ion)	Error (ppm)	References	μg/g of D.W.^#^
1	Dihydromyricetin	C_15_H_12_O_8_	10.07	319.0467	125.0235 (100), 151.0030 (38.63), 191.0347 (26.34), 193.0140 (57.87)	5.787	Standard	7975.01 ± 119.63
2	Iso-dihydromyricetin^a^	C_15_H_12_O_8_	10.67	319.0466	141.0296 (1.54), 151.0031 (3.80), 193.0140 (100)	5.317	[14,25,26]	4184.91 ± 89.56
3	Myricetin-*3*-*O*-glucoside	C_21_H_20_O_13_	10.76	479.0842	317.0285 (29.33), 316.0233 (100)	4.557	Standard	61.74 ± 1.54
4	Myricetin-*3*-*O*-rhamnoside	C_21_H_20_O_12_	11.71	463.0894	316.0232 (100), 300.0282 (58.44), 271.0254 (7.16)	5.005	Standard	1007.99 ± 32.25
5	Quercetin-3-*O*-xyloside^a^	C_20_H_18_O_11_	12.6	433.0789	300.0283 (100), 301.0338 (30.29)	5.570	[14], Massbank	2.89 ± 0.03
6	Quercetin-*3*-*O*-rhamnoside	C_21_H_20_O_11_	12.99	447.0944	300.0284 (100), 151.0026 (3.46)	5.194	Standard	89.79 ± 2.36
7	Quercetin	C_15_H_10_O_7_	13.07	301.0361	121.0285 (20.70), 124.0157 (100),	5.949	Standard	144.23 ± 1.96
8	Phloridzin	C_21_H_24_O_10_	13.77	435.1308	273.0777 (62.40), 167.0344 (100)	5.048	Standard	0.89 ± 0.03
9	Phloretin	C_15_H_14_O_5_	13.82	273.0777	167.03345 (39.56), 123.0442 (100), 125.0235 (20.10)	7.031	Standard	63.78 ± 1.61
10	Myricetin	C_15_H_10_O_8_	13.86	317.0309	125.0235 (100), 137.0235 (58.85), 151.0030 (92.58), 165.0188 (27.91)	5.319	Standard	788.55 ± 18.51

RT*: retention time; D.W.^#^: dry weight of extract; Iso-dihydromyricetin^a^ and quercetin-3-*O*-xyloside^a^ were semi-quantified by dihydromyricetin and quercetin-3-*O*-rhamnoside standards, respectively; the other eight compounds were quantified by their corresponding commercial standards.

**Table 2:** Results of color parameters of breads treated with or without vine tea extracts or dihydromyricetin.

Sample	Crust Color			Crumb Color		
	L* ^A^	a* ^B^	b* ^C^	L* ^A^	a* ^B^	b* ^C^
Control	37.45 ± 1.22 ^b^	16.51 ± 0.90 ^a^	24.02 ± 0.51 ^a^	62.68 ± 0.79 ^a^	1.66 ± 0.09 ^a^	16.50 ± 0.43 ^a^
Vine tea extract 1.25 g/kg	44.26 ± 0.87 ^a^	15.61 ± 0.60 ^a^	22.04 ± 0.33 ^b^	58.49 ± 1.30 ^b^	0.27 ± 0.05 ^c^	13.47 ± 0.23 ^c^
Vine tea extract 2.5 g/kg	44.52 ± 0.71 ^a^	11.17 ± 0.21 ^c^	18.77 ± 0.57 ^c^	58.24 ± 1.20 ^b^	0.45 ± 0.08 ^e^	14.86 ± 0.36 ^b^
Dihydromyricetin 9.97 mg/kg	43.93 ± 0.82 ^a^	14.74 ± 0.81 ^b^	25.44 ± 0.98 ^a^	62.54 ± 1.25 ^a^	0.12 ± 0.05 ^d^	16.27 ± 0.32 ^a^
Dihydromyricetin 19.94 mg/kg	38.54 ± 0.75 ^b^	10.01 ± 0.17 ^d^	24.43 ± 0.59 ^a^	62.36 ± 1.43 ^a^	1.04 ± 0.15 ^b^	15.79 ± 0.45 ^a^

L*^A^ = lightness; a*^B^ = redness; b*^C^ = yellowness. Values are expressed as the mean ± SD (*n* = 3); data in the same column without superscript letter (a, b, and c) in common differ significantly (*p* < 0.05).

**Table 3:** Texture profiles and moisture contents of bread crumb treated with or without vine tea extracts or dihydromyricetin.

Sample	Moisture (%)	Hardness (g)	Springiness	Cohesiveness	Gumminess (g)	Chewiness (g)	Resilience
Control	39.55 ± 0.57 ^a^	272.04 ± 1.93 ^a^	0.73 ± 0.02 ^b^	0.64 ± 0.04 ^a^	174.43 ± 4.21 ^a^	127.68 ± 4.88 ^a^	0.23 ± 0.05 ^a^
Vine tea extract 1.25 g/kg	39.40 ± 0.33 ^a^	141.60 ± 0.67 ^c^	0.81 ± 0.02 ^a^	0.65 ± 0.02 ^a^	92.39 ± 2.63 ^c^	75.39 ± 2.26 ^c^	0.22 ± 0.02 ^a^
Vine tea extract 2.5 g/kg	40.16 ± 0.44 ^a^	83.67 ± 1.72 ^d^	0.71 ± 0.01 ^b^	0.65 ± 0.01 ^a^	54.56 ± 3.75 ^d^	37.92 ± 2.25 ^e^	0.23 ± 0.03 ^a^
Dihydromyricetin 9.97 mg/kg	39.30 ± 0.58 ^a^	227.84 ± 0.75 ^b^	0.78 ± 0.02 ^a^	0.67 ± 0.01 ^a^	152.87 ± 3.06 ^b^	118.12 ± 2.01 ^b^	0.26 ± 0.03 ^a^
Dihydromyricetin 19.94 mg/kg	40.39 ± 0.62 ^a^	76.47 ± 1.58 ^e^	0.82 ± 0.01 ^a^	0.71 ± 0.01 ^a^	54.61 ± 0.78 ^d^	46.84 ± 1.57 ^d^	0.23 ± 0.01 ^a^

Values are expressed as the mean ± SD (*n* = 3). Data in the same column without superscript letter (a, b, and c) in common differ significantly (*p* < 0.05).

**Table 4:** The results of triangle and paired preference tests.

Triangle Tests			Paired Preference Test	
Quality Attributes	Number of Correct Responses	Significant Difference ^a^	Samples	Number of Acceptance Responses ^b^
Aroma	19/30	*p* < 0.01	Control	13
Appearance	26/30	*p* < 0.01	Treatment	17
Taste	16/30	*p* < 0.05		

^a^ Critical number of correct responses required for a statistical significance in a triangle test by referring to Yi et al. and Civille et al. [23,24]; ^b^ No significant difference of acceptability between the control bread and the sample bread with vine tea extract (*p* > 0.05) according to the statistical table of Civille et al. [24].
